# Evaluating perspectives and attitudes towards the South African medical internship programme

**DOI:** 10.1186/s12909-025-07994-y

**Published:** 2025-10-10

**Authors:** R. Boden, MI Majiet, I. Balde, S. Maswime

**Affiliations:** Global Surgery Division, Department of Surgery, Faculty of Health Sciences, University of Cape Town, Cape Town, South Africa

**Keywords:** Human resources for health, Medical education, Education, Health for all, Global Surgery, Global Health

## Abstract

South Africa’s healthcare system faces a quadruple burden of disease and marked social inequalities, necessitating an increased number of well-trained medical personnel. The internship period, a two-year programme where junior doctors transition from medical school to professional practice, is central to this. However, limited data exist on its effectiveness and suitability.

**Methods** This survey-based study explored the expectations and perceptions of the South African internship programme among stakeholders in the Western Cape province.

**Results** A total of 82 individuals responded, including 63 medical interns and 19 independent doctors and specialists (intern curators and heads of department). The majority of interns expected to gain procedural skills (n=57, 90%), diagnostic skills (n=56, 89%), and meaningful interaction with senior colleagues to enhance learning (n=53, 84%). Most interns felt their rotations prepared them for independent practice: family medicine (96%), anaesthesia (94%), psychiatry and mental health (86%), internal medicine (81%), paediatrics (76%), and obstetrics and gynaecology (71%). Only 38% felt surgery prepared them adequately. Respondents highlighted that shortening this rotation would further compromise readiness for independent practice.

Regarding internship length, 68% believed reducing the current two-year programme to one year would be harmful, while 9% perceived no harm and 13% were unsure. If shortened, 83% expected less clinical exposure and 75% anticipated reduced teaching time. A 24-month programme was consistently viewed as offering greater benefit for skills acquisition than any shorter alternative.

**Conclusion** The South African internship programme is largely regarded by interns and supervisors as effective in preparing junior doctors for independent practice. However, the surgery rotation is widely seen as insufficient. Acquisition of procedural and diagnostic skills remains a key priority and should continue to guide programme design. Most stakeholders favour maintaining the two-year structure, as it provides more substantial clinical exposure and training benefits than a shortened internship.

**Supplementary Information:**

The online version contains supplementary material available at 10.1186/s12909-025-07994-y.

## Background

South Africa’s health system faces a quadruple burden of disease: HIV/AIDS, Tuberculosis, maternal and child health, non-communicable diseases, trauma due to injury and violence [1] and an unequal society reflecting the legacy of apartheid and coloniality [2]. Interviews with healthcare users concluded that the number of health workers is ‘not sufficient for the number of patients’, further complicated by absenteeism, leave and compulsory training [3]. South Africa needs more adequately trained medical personnel, including doctors, to address these challenges.

In South Africa, medical doctors undergo undergraduate teaching at one of eight medical schools, followed by a two-year internship regulated by the Health Professions Council of South Africa (HPCSA) [4]. This internship period ensures a smooth transition from theoretical knowledge to practical skills for junior doctors to equip themselves to function as competent and safe medical practitioners. Medical internships are a common prerequisite for independent practice or registration for specialisation across the world, with countries like Ireland, Denmark and Saudi Arabia requiring a single-year internship and countries like Egypt, Chile and Ghana requiring completion of a two-year internship programme. In 2020, there were 43,901 registered medical doctors in South Africa, of which 4,458 were internship posts [4]. Medical interns make up approximately 10% of the total medical doctor workforce in the country. This highlights the importance of optimising the placement and training of medical interns to deliver high-quality health services to tackle challenges faced by the South African healthcare system.

While interns gain valuable teaching and practical experience during the two years, there are concerns regarding supervision during interventional procedures, with a national survey conducted by Bola et al. declaring a “significant failure in providing supervision of interns performing interventional procedures for the first time” [5]. The researchers hypothesise that this promotes a cycle of “confident incompetence” described by Marteau et al., where young doctors gain confidence in doing procedures without the necessary training and expertise, compromising patient safety in the process [6]. Generally, hospitals are responsible for planning and implementing training programmes for their interns, under the auspices of the Health Professions Council of South Africa. Therefore, the inter-facility variability is large when it comes to supervision, training and assessment. Certainly, in many hospitals, interns will act as health providers without the direct supervision of a senior, even when performing procedures. However, interns are, at all times, meant to be under the authority of a senior doctor. Seniors in this context refers to doctors who have completed internship and community service time and are registered with the HPCSA as independent practitioners. These senior doctors can be medical officers, registrars (specialists-in-training) or consultants (specialist physicians in a specific discipline) who will bear the responsibility for the interns’ clinical decisions and outcomes. Each cohort fulfils distinct roles within the clinical team, contributing both to patient care and to the intern’s learning, supervision, and professional development.

The decision to increase the length of the medical internship programme from one year to two years was made in 2005, with 2006 being the beginning of the first two-year programme cohort. The decision was made to ‘ensure that all medical graduates are adequately prepared to work at a district hospital during their year of community service’ and to address concerns regarding maternal mortality, clinical skills, and anaesthetic competency during the one-year programme [7].

There have been discussions about reviewing the length of medical internships in response to concerns about poor management, limited leadership, challenging work environments, and heavy workloads [8]. However, the South African Medical Association (SAMA) have publicly rejected all possible length changes and encouraged a shift towards district-level-of-care training and work in community health centres to decrease the shortage of internship posts as alternatives to the shortening of the programme [7]. The HPCSA announced, in their 2019 Annual Report, that the internship period will remain a two-year programme with extended training in primary health care [9].

Given the above, evaluating attitudes and perceptions around the internship programme is essential to improving the efficiency of our healthcare system. This study aimed to examine the perceptions of the internship programme and its suitability held by medical interns, intern curators and heads of departments in hospitals in the Western Cape province of South Africa.

## Methods

This was a cross-sectional descriptive study. A survey was distributed through online platforms to doctors across the Western Cape of South Africa working at hospitals connected to the University of Cape Town and Stellenbosch University - namely the following hospitals: Groote Schuur Hospital; Victoria Hospital; New Somerset Hospital; Mitchell’s Plain District Hospital; Red Cross Children’s Hospital; Tygerberg Hospital; Karl Bremer Hospital; Khayelitsha District Hospital. The survey was sent to doctors with the following job titles: Medical interns, community service doctors, medical officers, internship curators (senior doctors in hospitals across South Africa responsible for their hospital internship programme) and Heads of departments who have the role of supervising interns of the relevant disciplines (Internal medicine, Surgery, Obstetrics, Paediatrics, Anaesthesia, Family medicine and Psychiatry). The survey was hosted via RedCap with user password-protected logins to protect the data. The survey was anonymous, with no personal details shared with the authors except for the email addresses of respondents to ensure no duplication of results. These personal details were kept confidential by the authors.

The survey was divided into sections that focused on: demographics, experiences of the internship programme, perceived goals of the internship programme, perceptions around the length of the internship period and then the possible effects of changes to the internship programme. Most questions were asked to each stakeholder; however, some questions were specifically for current interns, especially those aiming to get an idea of the current state of the internship period. The survey was originally developed for this study specifically by the authorial team and is available as a supplementary file. Data collection was done from June 2022 to September 2022; thus, the interns who answered the survey were first or second-year interns during the 2022 calendar year. Descriptive summaries were used throughout the analysis, the chi-square test was used to test categories and the fisher’s-exact to test categories when cell counts were below 5.

The study was approved by the University of Cape Town’s Human Research Ethics Committee as Project 2021/180.

## Results

A total of 82 individuals responded to the survey: 36 first-year interns, 27 s-year interns (63 interns in total), 13 Heads of Department (HODs), and 4 internship curators. Two additional respondents were excluded as they were specialists not serving as HODs or curators. Internship curators may be specialists and, in some cases, also HODs; where this overlap occurred, responses were counted under the curator category only. All 82 eligible responses were included in the analysis. Results are reported by cohort according to the questions posed.

In South Africa, internship programmes are hospital-based and not centralised, with no publicly available databases from which to estimate the total number of possible participants. For this reason, the generalisability of findings beyond the study respondents cannot be determined.

### Expected gains from the internship period

Interns most frequently identified the following as key expectations: acquisition of procedural skills (*n* = 57, 90%), diagnostic skills (*n* = 56, 89%), opportunities to interact with senior colleagues to support learning and skills development (*n* = 53, 84%), confidence in managing patients independently (*n* = 53, 84%), and immediate access to supervisory support when needed (*n* = 51, 81%).

The least-selected expectations included improvement of time management skills (*n* = 24, 38%), opportunities to explore new areas of the country (*n* = 11, 17%), involvement in or completion of research projects (*n* = 11, 17%), access to subsidised accommodation (*n* = 5, 8%), and other motivations (*n* = 2, 3%) (Tables 1 and 2).

**Table 1:** Expected gains from the internship period

What did you expect to gain from your internship time?	Overall *N* = 63	First Years *N* = 36	Second Years *N* = 27
Procedural skills	57 (90%)	34 (94%)	23 (85%)
Diagnostic skills	56 (89%)	33 (92%)	23 (85%)
Opportunity to interact with seniors in ways that promote your learning and skills development	53 (84%)	27 (75%)	26 (96%)
Confidence in managing patients independently	53 (84%)	30 (83%)	23 (85%)
Immediate help from relevant supervisor should this be needed	51 (81%)	28 (78%)	23 (85%)
Relevant and helpful feedback from supervisors regularly	50 (79%)	29 (81%)	21 (78%)
Experience and support in practising medicine that is evidence-based	48 (76%)	25 (69%)	23 (85%)
Learning how to prioritise patient safety - specifically through teaching by and the example of clinical supervisors)	47 (75%)	24 (67%)	23 (85%)
Appropriate induction into your role and the hospital you are placed in	45 (71%)	23 (64%)	22 (81%)
A time of definite job security	43 (68%)	27 (75%)	16 (59%)
More clinical responsibility	40 (63%)	20 (56%)	20 (74%)
A chance to build relationships with other health professionals	39 (62%)	21 (58%)	18 (67%)
A good salary with comfortable living option	38 (60%)	19 (53%)	19 (70%)
Surgical experience	36 (57%)	22 (61%)	14 (52%)
Experience in prioritising patient safety	30 (48%)	11 (31%)	19 (70%)
Time management skills	24 (38%)	13 (36%)	11 (41%)
An opportunity to explore a new area of the country	20 (32%)	13 (36%)	7 (26%)
To be involved in or complete research projects	11 (17%)	5 (14%)	6 (22%)
Access to subsidised accommodation (doctors quarters)	5 (8%)	3 (8.3%)	2 (7.4%)
Other	2 (3%)	2 (5.6%)	0 (0%)

**Table 2:** Where do interns feel prepared to practise?

	Overall *N* = 63	First Years *N* = 36	Second Years *N* = 27
What level of care would you prefer to spend the bulk of your internship practising in?			
District hospital	10 (16%)	5 (14%)	5 (19%)
Primary care clinic	3 (4.8%)	1 (2.8%)	2 (7.4%)
Secondary level academic hospital	20 (32%)	10 (28%)	10 (37%)
Tertiary level academic hospital	4 (6.3%)	2 (5.6%)	2 (7.4%)
A mix of the above	26 (41%)	18 (50%)	8 (30%)
At which level of care do you see yourself spending the bulk of your future practising time?			
District hospital	8 (13%)	4 (11%)	4 (15%)
Primary care clinic	7 (11%)	4 (11%)	3 (11%)
Secondary level academic hospital	10 (16%)	4 (11%)	6 (22%)
Tertiary level academic hospital	16 (25%)	7 (19%)	9 (33%)
A mix of the above	22 (35%)	17 (47%)	5 (19%)
Where do you think practising during internship would make you best prepared for community service and independent practice?			
Rural setting	1 (1.6%)	0 (0%)	1 (3.7%)
Suburban setting	12 (19%)	6 (17%)	6 (22%)
Urban setting	9 (14%)	8 (22%)	1 (3.7%)
A mix of the above	41 (65%)	22 (61%)	19 (70%)
Where would you prefer to spend the bulk of your internship time?			
Peri-urban setting	13 (21%)	8 (22%)	5 (19%)
Rural setting	1 (1.6%)	0 (0%)	1 (3.7%)
Urban setting	32 (51%)	17 (47%)	15 (56%)
A mixture of the above	17 (27%)	11 (31%)	6 (22%)
Where do you currently feel least prepared to practice?			
District hospital	7 (11%)	6 (17%)	1 (3.7%)
Primary care clinic	24 (38%)	17 (47%)	7 (26%)
Secondary level academic hospital	8 (13%)	2 (5.6%)	6 (22%)
Tertiary level academic hospital	24 (38%)	11 (31%)	13 (48%)

### Level of care preferences

Interns are placed across South Africa during their internship period and gain exposure to a range of healthcare settings. The majority (*n* = 41, 65%) believed that training in a mixed environment (urban, semi-urban, and rural) would best prepare them for community service and independent practice. Despite this, 51% still preferred to spend most of their internship in an urban setting. In terms of levels of care, 41% (*n* = 26) favoured training across a mix of levels, while 32% (*n* = 20) preferred secondary-level care.

### Intern perception of training adequacy

Overall, interns felt that rotations prepared them for independent practice. As shown in Table 3 and 96% reported being well prepared by family medicine, 94% by anaesthesia, 86% by psychiatry and mental health, 81% by internal medicine, 76% by paediatrics, 71% by obstetrics and gynaecology, and 38% by surgery (Table 4).

**Table 3:** Does internship prepare interns to practise independently in South africa??

Do you feel like your training and exposure in the following disciplines during your internship programme has been/was adequate for you to become an independent medical practitioner in South Africa?			
Discipline	Overall *N* = 84	First Years *N* = 36	Second Years *N* = 27
Internal Medicine			
Yes	39 (81%)	17 (81%)	22 (81%)
No	9 (19%)	4 (19%)	5 (19%)
(Missing)	36	15	0
Surgery			
Yes	21 (38%)	11 (38%)	10 (37%)
No	35 (62%)	18 (62%)	17 (63%)
(Missing)	28	7	0
Obstetrics and Gynaecology			
Yes	36 (71%)	15 (62%)	21 (78%)
No	15 (29%)	9 (38%)	6 (22%)
(Missing)	33	12	0
Paediatrics			
Yes	34 (76%)	15 (83%)	19 (70%)
No	11 (24%)	3 (17%)	8 (30%)
(Missing)	39	18	0
Psychiatry and mental health			
Yes	12 (86%)	0 (NA%)	12 (86%)
No	2 (14%)	0 (NA%)	2 (14%)
(Missing)	70	36	13
Anaesthesia			
Yes	15 (94%)	0 (NA%)	15 (94%)
No	1 (6.2%)	0 (NA%)	1 (6.2%)
(Missing)	68	36	11
Family medicine			
Yes	24 (96%)	0 (NA%)	24 (96%)
No	1 (4.0%)	0 (NA%)	1 (4.0%)
(Missing)	59	36	2

**Table 4:** Do interns perceive that they are well-supported in South africa??

	Overall *N* = variable	First Years *N* = 36	Second Years *N* = 27
Do you feel like you have been adequately supported/supervised during your internship programme? *N* = 84			
Yes	53 (84%)	30 (83%)	23 (85%)
No	10 (16%)	6 (17%)	4 (15%)
(Missing)	21	0	0
Which department below do you feel you had the most support or supervision? (Only first-year respondents, as these rotations are only done in the first year of internship) *N* = 36			
Medicine	8 (22%)	8 (22%)	N/A
Obstetrics and Gynaecology	16 (44%)	16 (44%)	
Paediatrics	9 (25%)	9 (25%)	
Surgery	3 (8.3%)	3 (8.3%)	
(Missing)	48	0	27
Which department below do you feel gave you the most support or supervision? (Only second-year respondents as some of these rotations are only done in the second year of internship i.e. Family medicine, anaesthesia and psychiatry) *N* = 27			
Anaesthesia	11 (41%)	N/A	11 (41%)
Family medicine	2 (7.4%)		2 (7.4%)
Medicine	4 (15%)		4 (15%)
Obstetrics and Gynaecology	6 (22%)		6 (22%)
Paediatrics	2 (7.4%)		2 (7.4%)
Psychiatry	1 (3.7%)		1 (3.7%)
Surgery	1 (3.7%)		1 (3.7%)
Do you feel that the logbook requirements mandated for interns during their internship programme adequately address the public health needs of South Africa? *N* = 84			
Yes	37 (59%)	20 (56%)	17 (63%)
No	26 (41%)	16 (44%)	10 (37%)
(Missing)	21	0	0
Do you feel that the logbook requirements for interns during their internship programme adequately prepare interns to become independent medical practitioners in South Africa? *N* = 84			
Yes	42 (67%)	23 (64%)	19 (70%)
No	21 (33%)	13 (36%)	8 (30%)
(Missing)	21	0	0

### Perceptions of supervision and support

Table 4 shows that most interns (84%) felt adequately supported and supervised. First-year interns reported the highest levels of support in obstetrics and gynaecology (44%), followed by paediatrics (25%), internal medicine (22%), and surgery (8.3%). Among second-year interns, support was perceived as strongest in anaesthesia (41%), obstetrics and gynaecology (22%), internal medicine (15%), paediatrics and family medicine (both 7.4%), psychiatry (3.7%), and surgery (3.7%).

Regarding the HPCSA Internship logbook, a document intended to collate the minimum core competencies and procedures required for an intern to be promoted to an independent practitioner, 59% agreed that requirements align with South Africa’s needs, while 41% disagreed. Two-thirds (67%) believed the logbook would prepare them for independent practice, compared with 33% who disagreed.

### Impact of shortened internship

Respondents highlighted concerns about reducing the internship to one year. While 35% thought it might allow a quicker transition to independent practice, 65% believed this would negatively impact readiness. Just over half (54%) felt a shortened period would reduce mandatory time in the public sector, with 66% viewing this as a flaw. Similarly, 62% thought there would be less time before registrar training, and of those who agreed there would be less time before registrar training, 68% considered this a disadvantage.

75% of responders believed shortening the internship period would harm their surgical training, and 74% would oppose shortening of the internship program if their surgical training was affected. Regarding minimum standards of care, only 25% thought a one-year programme would provide enough training and exposure to maintain safety and professional development for obstetrics, and 19% for both surgery and anaesthesia. Overall, 11% believed a shortened internship could still produce safe doctors, while 19% stated that this was possible only with specific provisions. In contrast, 56% believed a one-year internship could not produce safe doctors in South Africa.

When asked about preferred internship duration, 75% supported the current two-year model, 18% favoured one year, and 7% preferred three years.

### Potential harms of a shortened internship period

Table 5 shows that 68% of respondents believed a shortened internship would cause harm, 9% felt it would not, and 13% were unsure. Most anticipated reduced clinical exposure (83%) and decreased teaching time (75%). Nearly half expected fewer mentorship opportunities (48%) and less job security (46%).

**Table 5:** *What are the perceptions around a shortened internship period?* Each individual question is in bold

Characteristic	Overall *N* = Variable	First Years *N* = 36	Second Years *N* = 27
**Do you think any harm would be associated with a shortened internship period?** *N* = 57			
Yes	39 (68%)	22 (76%)	16 (62%)
No	7 (9%)	3 (10%)	3 (12%)
Unsure	11 (13%)	4 (14%)	7 (27%)
(Missing)	25	7	1
**What harms do you think would be associated with a shortened internship period?** N = 63			
a. None			
Yes	3 (4.7%)	2 (3.1%)	0 (0%)
No			
b. Inadequate teaching time			
Yes	47 (75%)	25 (69%)	21 (78%)
No			
c. Decreased mentorship opportunities			
Yes	30 (48%)	15 (42%)	14 (52%)
No			
d. Job security for less time			
Yes	29 (46%)	15 (42%)	13 (48%)
No			
e. Decreased clinical exposure			
Yes	52 (83%)	27 (75%)	24 (89%)
No			
f. Decreased chance of doing research			
Yes	11 (17%)	3 (8.3%)	7 (26%)
No			
g. Increase in competition in private sector			
Yes	11 (17%)	8 (22%)	3 (11%)
No			
h. Increased competition for further training			
Yes	20 (32%)	12 (33%)	7 (26%)
No			
**Do you think a shortened internship period should be optional? i.e. should prospective interns be able to choose between a one- and two-year internship?** *N* = 57			
Yes	13 (23%)	6 (21%)	6 (23%)
Yes - but only if there is a valid reason to choose a shortened period, i.e. academic advancement, family emergencies	14 (25%)	9 (31%)	5 (19%)
No	29 (51%)	13 (45%)	15 (58%)
Unsure	1 (1.8%)	1 (3.4%)	0 (0%)
**Do you think that there should be flexibility in internship training in terms of what rotations and specialities you choose to complete in 1 or 2 years and for what lengths?** *N* = 73			
Yes	48 (66%)	21 (72%)	19 (73%)
No	23 (32%)	6 (21%)	7 (27%)
Unsure	2 (2.7%)	2 (6.9%)	0 (0%)
**Do you think the possible benefits of a shortened internship outweigh the possible disadvantages?** *N* = 57			
Yes	11 (19%)	5 (17%)	5 (19%)
No	35 (61%)	18 (62%)	16 (62%)
Unsure	11 (19%)	6 (21%)	5 (19%)
**Do you think a one-year internship period is preferable to a two-year period on a national, mandated level?** *N* = 57			
Benefits probably outweigh potential harms	3 (5.3%)	1 (3.4%)	2 (7.7%)
Definitely	5 (8.8%)	2 (6.9%)	2 (7.7%)
Definitely not	23 (40%)	10 (34%)	12 (46%)
Either is acceptable	8 (14%)	4 (14%)	4 (15%)
There are benefits, but not enough to warrant this change	18 (32%)	12 (41%)	6 (23%)
**Do you think a truncated internship period would lead to the general public having mistrust in the health system or competency of doctors?** *N* = 57			
Yes	22 (39%)	11 (38%)	10 (38%)
Yes - unless specific information and campaigning is done to allay fears	11 (19%)	7 (24%)	4 (15%)
No	15 (26%)	8 (28%)	6 (23%)
Unsure	9 (16%)	3 (10%)	6 (23%)
**Do you think a 1-year internship would compromise patient care during the internship period?** *N* = 57			
Yes	31 (54%)	14 (48%)	16 (62%)
No	19 (33%)	10 (34%)	8 (31%)
Unsure	7 (12%)	5 (17%)	2 (7.7%)
**Do you think a 1-year internship would compromise patient care by the interns that have 1 year after they finish their internship?** *N *= 57			
Yes	35 (61%)	16 (55%)	18 (69%)
No	11 (19%)	6 (21%)	4 (15%)
Unsure	11 (19%)	7 (24%)	4 (15%)
**Do you think shortening the period to one year would result in greater supervision of interns as they provide patient care?** *N *= 57			
Yes	15 (26%)	8 (28%)	6 (23%)
No	36 (63%)	17 (59%)	18 (69%)
Unsure	6 (11%)	4 (14%)	2 (7.7%)
**Do you think a one-year internship would give interns the minimum competencies required for safe surgical care?** *N *= 57			
Yes	11 (19%)	6 (21%)	4 (15%)
No	31 (54%)	13 (45%)	17 (65%)
Unsure	15 (26%)	10 (34%)	5 (19%)
**Do you think a one-year internship would give interns the minimum competencies required for obstetric care?** *N* = 57			
Yes	14 (25%)	9 (31%)	5 (19%)
No	33 (58%)	14 (48%)	18 (69%)
Unsure	10 (18%)	6 (21%)	3 (12%)
**Do you think a one-year internship would give interns the minimum competencies required for safe anaesthesia care?** *N *= 57			
Yes	11 (19%)	5 (17%)	5 (19%)
No	37 (65%)	18 (62%)	18 (69%)
Unsure	9 (16%)	6 (21%)	3 (12%)
**Do you think a one-year internship would give interns the minimum competencies required for overall patient care as an independent medical practitioner in South Africa?** *N *= 57			
Yes	6 (11%)	2 (6.9%)	3 (12%)
Yes, but with conditions	11 (19%)	8 (28%)	3 (12%)
No	32 (56%)	16 (55%)	15 (58%)
Unsure	8 (14%)	3 (10%)	5 (19%)
**What duration of internship would you, personally, prefer considering all of the above-mentioned possible benefits and flaws?** *N *= 57			
One year	10 (18%)	4 (14%)	5 (19%)
Three years	4 (7.0%)	2 (6.9%)	2 (7.7%)
Two years	43 (75%)	23 (79%)	19 (73%)

55% of respondents believed patient care during internship would be compromised by a one-year programme, while 33% felt it would not, and 12% were unsure. Regarding care after internship, 62% believed quality of care would be affected, 19% were unsure, and 19% thought care would remain unaffected.

On supervision, 63% did not expect a shortened period to increase supervisory support, while only 26% anticipated more. When weighing potential benefits against disadvantages, 62% concluded the disadvantages outweighed any gains, 19% felt the opposite, and 19% were uncertain.

### Perceived priorities of the internship period

Tables 6, 7 and 8 highlights stakeholders’ views on the programme’s priorities for producing safe, independent practitioners. The most frequently identified priority was diagnostic skills training (84%), followed by procedural skills (64%), confidence in independent patient management (61%), and emphasis on patient safety through teaching and role modelling by supervisors (48%). Experience and support in evidence-based medicine were selected by 46%. The least valued priorities were access to subsidised accommodation (2%) and opportunities to explore a new area of the country (4%).

**Table 6:** Potential ramifications of a shorter internship period

Characteristic	*N*	Overall *N* = 82	First Years *N* = 36	Second Years *N* = 27	Community service doctor *N* = 1
Do you think any of the following situations are possible if the internship period is shortened to a single year?					
Interns will have more freedom of choice in living situation	82				
Yes		11 (13%)	6 (17%)	4 (15%)	1 (100%)
No		71 (87%)	30 (83%)	23 (85%)	0 (0%)
Interns will have a quicker transition to independent medical practice)	82				
Yes		29 (35%)	16 (44%)	12 (44%)	1 (100%)
No		53 (65%)	20 (56%)	15 (56%)	0 (0%)
Interns will have less mandatory time in the public sector	82				
Yes		44 (54%)	24 (67%)	19 (70%)	1 (100%)
No		38 (46%)	12 (33%)	8 (30%)	0 (0%)
Interns will have less time before registrar training	82				
Yes		51 (62%)	29 (81%)	21 (78%)	1 (100%)
No		31 (38%)	7 (19%)	6 (22%)	0 (0%)
Do you think that interns having more freedom of choice in living circumstances would be a benefit of a shortened internship period or a flaw in a shortened internship period?	11				
Benefit		9 (82%)	4 (67%)	4 (100%)	1 (100%)
Flaw		1 (9.1%)	1 (17%)	0 (0%)	0 (0%)
Unsure		1 (9.1%)	1 (17%)	0 (0%)	0 (0%)
(Missing)		71	30	23	0
Do you think that interns having a quicker transition to independent practice would be a benefit of a shortened internship period or a flaw in a shortened internship period?	28				
Benefit		9 (32%)	4 (27%)	4 (33%)	1 (100%)
Flaw		18 (64%)	11 (73%)	7 (58%)	0 (0%)
Unsure		1 (3.6%)	0 (0%)	1 (8.3%)	0 (0%)
(Missing)		54	21	15	0
Do you think that interns having less mandatory time in the public sector would be a benefit of a shortened internship period or a flaw in a shortened internship period?	44				
Benefit		10 (23%)	5 (21%)	4 (21%)	1 (100%)
Flaw		29 (66%)	16 (67%)	13 (68%)	0 (0%)
Unsure		5 (11%)	3 (12%)	2 (11%)	0 (0%)
(Missing)		38	12	8	0
Do you think that interns having less time before further training would be a benefit of a shortened internship period or a flaw in a shortened internship period?	50				
Benefit		15 (30%)	8 (29%)	6 (29%)	1 (100%)
Flaw		34 (68%)	20 (71%)	14 (67%)	0 (0%)
Unsure		1 (2.0%)	0 (0%)	1 (4.8%)	0 (0%)
(Missing)		32	8	6	0
Please click on what you think the top 5 priorities for the internship period would be if the goal of the internship programme were to create safe independent medical practitioners					
A good salary with comfortable living options	56				
Checked		12 (21%)			
Unchecked		44 (79%)			
Appropriate induction into your role and the hospital you are placed in	56				
Checked		22 (39%)			
Unchecked		34 (61%)			
Surgical experience	56				
Checked		23 (41%)			
Unchecked		33 (59%)			
Diagnostic skills	56				
Checked		47 (84%)			
Unchecked		9 (16%)			
Opportunity to interact with seniors in ways that promote your learning and skills development	56				
Checked		17 (30%)			
Unchecked		39 (70%)			
Experience in prioritising patient safety	56				
Checked		23 (41%)			
Unchecked		33 (59%)			
Learning how to prioritise patient safety - specifically through teaching by and the example of clinical supervisors	56				
Checked		27 (48%)			
Unchecked		29 (52%)			
Relevant and helpful feedback from supervisors on a regular basis	56				
Checked		23 (41%)			
Unchecked		33 (59%)			
Immediate help from relevant supervisor should this be needed	56				
Checked		21 (38%)			
Unchecked		35 (62%)			
Experience and support in practising medicine that is evidence-based	56				
Checked		26 (46%)			
Unchecked		30 (54%)			
To be involved in or complete research projects	56				
Checked		7 (13%)			
Unchecked		49 (87%)			
Procedural skills	56				
Checked		36 (64%)			
Unchecked		20 (36%)			
A chance to build relationships with other health professionals	56				
Checked		10 (18%)			
Unchecked		46 (82%)			
Time management skills	56				
Checked		11 (20%)			
Unchecked		45 (80%)			
An opportunity to explore a new area of the country	56				
Checked		2 (4%)			
Unchecked		54 (96%)			
Access to subsidised accommodation (doctors quarters)	56				
Checked		1 (2%)			
Unchecked		55 (98%)			
A time of definite job security	56				
Checked		7 (13%)			
Unchecked		49 (87%)			
Confidence in managing patients independently	56				
Checked		34 (61%)			
Unchecked		22 (39%)			
More clinical responsibility	56				
Checked		10 (18%)			
Unchecked		46 (82%)			
Do you think a shortened internship would have a negative impact on interns’ surgical training?	57				
Checked		43 (75%)	22 (76%)	20 (77%)	0 (0%)
Unchecked		11 (19%)	5 (17%)	5 (19%)	1 (100%)
Unsure		3 (5.3%)	2 (6.9%)	1 (3.8%)	0 (0%)
Unanswered		25	7	1	0
If you had to choose at the beginning of your internship: Would you be willing to compromise your surgical training in exchange for a shortened internship period?	43				
Checked		9 (21%)	4 (18%)	5 (25%)	0 (NA%)
Unchecked		32 (74%)	17 (77%)	14 (70%)	0 (NA%)
Unsure		2 (4.7%)	1 (4.5%)	1 (5.0%)	0 (NA%)
Unanswered		39	14	7	1
Do you think a shortened internship will harm interns’ obstetrics training?	57				
Checked		43 (75%)	21 (72%)	21 (81%)	0 (0%)
Unchecked		13 (23%)	7 (24%)	5 (19%)	1 (100%)
Unsure		1 (1.8%)	1 (3.4%)	0 (0%)	0 (0%)
Unanswered		25	7	1	0
If you had to choose at the beginning of your internship: Would you be willing to compromise your obstetrics training in exchange for a shortened internship period?	43				
Checked		7 (16%)	5 (24%)	2 (9.5%)	0 (NA%)
Unchecked		31 (72%)	15 (71%)	15 (71%)	0 (NA%)
Unsure		5 (12%)	1 (4.8%)	4 (19%)	0 (NA%)
Unanswered		39	15	6	1
Do you think a shortened internship will harm interns’ medical training?	57				
Checked		43 (75%)	24 (83%)	18 (69%)	0 (0%)
Unchecked		11 (19%)	4 (14%)	6 (23%)	1 (100%)
Unsure		3 (5.3%)	1 (3.4%)	2 (7.7%)	0 (0%)
Unanswered		25	7	1	0
If you had to choose at the beginning of your internship: Would you be willing to compromise your medical training in exchange for a shortened internship period?	43				
Unchecked		41 (95%)	24 (100%)	16 (89%)	0 (NA%)
Unsure		2 (4.7%)	0 (0%)	2 (11%)	0 (NA%)
Unanswered		39	12	9	1

Branched questions were only asked to the respondents who clicked ‘Yes’ to questions surrounding the internship period – thus many questions will have ‘missing’ respondents, those who did not answer yes to the original branching question. For example, one of the questions reads ‘Do you think any of the following situations are possible if the internship period is shortened to a single year? Interns will have more freedom of choice in living situation’. For this question, 11 answered yes. These 11 respondents were then asked a follow-up branched question ‘Do you think that interns having more freedom of choice in living circumstances would be a benefit of a shortened internship period or a flaw in a shortened internship period?’ which then had further answers

**Table 7:** What are the potential impacts of a shortened internship period?

	Overall *N* = Variable (84)	First Years *N* = 36	Second Years *N* = 27	Community Service Doctor *N* = 1	Head of Department *N* = 13	Internship Programme Curator *N* = 4
Do you think that a shortened internship period would place more pressure on hospital and intern managers? *N* = 76						
Yes	52 (68%)	20 (69%)	17 (65%)	0 (0%)	9 (69%)	3 (75%)
No	16 (21%)	6 (21%)	5 (19%)	1 (100%)	3 (23%)	1 (25%)
Unsure	8 (11%)	3 (10%)	4 (15%)	0 (0%)	1 (7.7%)	0 (0%)
(Missing)	8	7	1	0	0	0
Do you think that a shortened internship period would place undue pressure on interns? *N* = 74						
Yes	52 (70%)	22 (76%)	18 (69%)	0 (0%)	8 (62%)	3 (75%)
No	13 (18%)	5 (17%)	4 (15%)	1 (100%)	2 (15%)	1 (25%)
Unsure	9 (12%)	2 (6.9%)	4 (15%)	0 (0%)	3 (23%)	0 (0%)
(Missing)	10	7	1	0	0	0
Do you think that a shortened internship would increase patient care and management at affected hospitals? *N* = 74						
Yes	49 (66%)	20 (69%)	16 (62%)	0 (0%)	9 (69%)	3 (75%)
No	13 (18%)	5 (17%)	3 (12%)	1 (100%)	3 (23%)	1 (25%)
Unsure	12 (16%)	4 (14%)	7 (27%)	0 (0%)	1 (7.7%)	0 (0%)
(Missing)	10	7	1	0	0	0

**Table 8:** What do intern curators think the priorities of the internship period should be?

Characteristic	Internship programme curator *N* = 4
Please click on what you think should be the top 5 priorities for the internship period if the goal of the programme was to develop competent independent medical practitioners	
A good salary with comfortable living options	
Unchecked	4 (100%)
Appropriate induction into your role and the hospital you are placed in	
Checked	2 (50%)
Unchecked	2 (50%)
Surgical experience	
Unchecked	4 (100%)
Diagnostic skills	
Checked	4 (100%)
Opportunity to interact with seniors in ways that promote your learning and skills development	
Checked	1 (25%)
Unchecked	3 (75%)
Experience in prioritising patient safety	
Checked	1 (25%)
Unchecked	3 (75%)
Learning how to prioritise patient safety - specifically through teaching by and the example of clinical supervisors	
Checked	2 (50%)
Unchecked	2 (50%)
Relevant and helpful feedback from supervisors on a regular basis	
Checked	1 (25%)
Unchecked	3 (75%)
Immediate help from relevant supervisor should this be needed	
Checked	1 (25%)
Unchecked	3 (75%)
Experience and support in practising medicine that is evidence-based	
Checked	2 (50%)
Unchecked	2 (50%)
To be involved in or complete research projects	
Unchecked	4 (100%)
Procedural skills	
Checked	3 (75%)
Unchecked	1 (25%)
A chance to build relationships with other health professionals	
Unchecked	4 (100%)
Time management skills	
Checked	2 (50%)
Unchecked	2 (50%)
An opportunity to explore a new area of the country	
Unchecked	4 (100%)
Access to subsidised accommodation (doctors quarters)	
Unchecked	4 (100%)
A time of definite job security	
Unchecked	4 (100%)
Confidence in managing patients independently	
Checked	1 (25%)
Unchecked	3 (75%)
More clinical responsibility	
Unchecked	4 (100%)

### Perceptions surrounding a shortened internship period

Table 5 shows that 52% of interns did not believe a shortened internship should be optional, compared with 23% who felt it should be available to all interns and 25% who supported it only under extenuating circumstances. Most interns (66%) endorsed flexibility in training regarding rotations and specialities, while 32% disagreed. Overall, 62% believed the potential harms of a shortened internship outweighed its benefits; 19% were unsure, and 19% felt benefits outweighed harms.

Concerns about patient care were prominent. 54% believed a shortened internship would decrease the quality of care during training, and 61% thought it would compromise future care delivered by interns completing an abridged programme. In contrast, 33% did not believe patient care during internship would be compromised, and 19% felt future care would remain unaffected.

Views on minimum competencies were cautious: 19% believed a truncated period could provide safe surgical training (54% disagreed), 25% thought it could suffice for obstetric care (58% disagreed), and 19% for anaesthesia (65% disagreed).

When asked about overall preparedness, 56% of interns felt a one-year internship would not provide minimum competencies for independent practice. In contrast, 11% believed it would, 19% thought it could under certain conditions, and 14% were unsure.

In terms of preferred duration, most interns (75%) supported maintaining a two-year internship, 18% preferred a single year, and 7% favoured extending it to three years.

### Intern curators’ perceptions

Intern curators think the most important priorities for the internship period should be diagnostic skills (100%), procedural skills (75%), appropriate inductions for interns (50%), time management skills (50%), experience and support in practising medicine that is evidence-based (50%), learning how to prioritise patient safety - specifically through teaching by and the example of clinical supervisors (50%). Some things that none of the curators thought should be a priority include surgical experience, a good salary, more clinical responsibility, and for interns to be involved in research projects during their internship.

## Discussion

Medical interns form a large cohort of South Africa’s health professionals, and they ‘must be acknowledged as an important part of the health workforce, contributing significantly to the functioning of the health system across the country’ [10]. This study analyses stakeholders’ perceptions of the internship programme.

This study found that interns generally felt well-prepared for independent practice by their rotations, except for the surgery rotation. Procedural and diagnostic skills were most frequently identified as essential outcomes, reflecting the importance of acquiring these competencies before community service, which is often undertaken in rural settings with limited specialist support.

While most interns believed that training across mixed settings (urban, semi-urban, and rural) best prepares them for community service, many still preferred spending the majority of their internship in urban hospitals. This preference likely reflects the availability of amenities, structured teaching, and specialist supervision in academic centres, which facilitate the acquisition of procedural and diagnostic skills. Interns highlighted the value of adequate supervision alongside opportunities for independent decision-making. They expected meaningful interaction with senior colleagues, immediate support when required, and regular feedback. These findings underscore the dual need for autonomy and supervision, with patient safety maintained by the presence of senior doctors such as medical officers, registrars, and consultants. Interactions with these clinicians provide essential learning opportunities while ensuring accountability and oversight. This desire is reflected by the preference of most interns to spend their programme at district or secondary hospitals in urban centres where there is typically closer supervision coupled with sufficient opportunity for skill development.

Overall, the results emphasise that supervision and skills acquisition are inseparable priorities of the South African internship programme. Future revisions must preserve both elements to ensure interns graduate with the competence and confidence required for independent clinical practice.

Interns reported that most rotations prepared them for independent practice, except for surgery. Family medicine, the longest rotation at six months, was particularly valued, as it provides exposure to multiple levels of care and opportunities to develop referral skills, minor surgical, and diagnostic skills under resource-limited conditions. This sense of preparedness is significant for the South African health system, where most patients first access care through primary healthcare clinics before referral to higher levels of expertise as needed. In contrast, surgery was not seen as adequate preparation for independent practice, and respondents believed a shortened rotation would further compromise readiness.

Support and supervision emerged as important strengths of the programme, with most interns reporting adequate oversight. First-year interns felt best supported in Obstetrics and Gynaecology, while second-year interns identified Anaesthesia, likely reflecting the structured nature of skills training in these fields. Both disciplines emphasise safety and competence, equipping interns for practice in rural settings where specialist support may be limited. Many anaesthesia departments reinforce this with pre-rotation assessments to identify knowledge gaps and post-rotation evaluations to ensure growth and readiness for independent practice.

Interns anticipate practising in a range of environments in their future, though most expressed a preference for specialised or academic centres, with only one reporting interest in rural practice. This contrasts with their perceived competence: nearly all interns felt well prepared by their family medicine training for independent practice, including in resource-limited rural settings. The disconnect suggests that while the internship programme equips doctors with skills for primary care, external factors—such as housing, safety, schooling, and transport—likely deter doctors from working in rural hospitals. This reflects longstanding trends in South Africa and internationally [11].

Perceptions of preparedness also varied by level of training. First-year interns most frequently reported feeling least prepared for primary care, while second-year interns were more confident in this domain. The increased exposure to family medicine in the second year likely contributes to this difference, underscoring the value of extended rotations in building competence. These findings indicate that direct experience within a discipline is key to improving confidence and preparedness, a principle relevant across all levels of care.

South Africa’s growing population, coupled with limited opportunities for specialisation, places increasing responsibility for patient care on junior doctors. This challenge is intensified by the rising prevalence of medically complex patients with multiple comorbidities, driven in part by improved life expectancy following widespread antiretroviral therapy adoption. Concurrently, the medical specialists per-capita is declining. Against this backdrop, robust exposure to family medicine and other foundational disciplines during internship is essential to prepare junior doctors for independent practice across diverse healthcare settings.

Most respondents felt the internship adequately prepared them for independent practice, with surgery being the exception. Limited exposure to surgical procedures during training likely explains this gap, which is concerning given South Africa’s significant surgical burden and reliance on non-specialists in rural and district hospitals. For many junior doctors, internship represents their only formal surgical training, underscoring the importance of maximising its effectiveness.

Proposals to shorten the internship are therefore problematic. Reduced training time would likely diminish competence, particularly in procedural fields such as surgery and anaesthesia. Compared with international norms, South Africa’s two-year internship provides broad clinical exposure and early responsibility, aligning with national healthcare needs and workforce shortages. The preference among most respondents for retaining the two-year format highlights the importance of preserving both its breadth and duration.

Overall, 68% of respondents believe a shortened programme would cause harm, while only 9% perceive no harm. 72% oppose shortening the internship, with just 14% supporting it. Differences in opinion reflect divergent views on the goals of internship: those prioritising procedural and diagnostic skills strongly oppose a shorter programme, while those favouring optional rotations or earlier specialisation are more supportive. Ultimately, shortening the internship risks undermining preparedness for safe, independent practice in a health system heavily dependent on broadly trained junior doctors.

Only 25% of respondents felt that a one-year internship would ensure minimum standards in obstetric care, and just 19% for surgery and anaesthesia. More than half (56%) believed a shortened internship could not produce safe doctors in South Africa. These findings provide a clear warning to policymakers: interns themselves perceive at least two years of training as necessary to ensure safe, independent practice.

International comparisons highlight both similarities and contrasts. In Saudi Arabia, a study by Swaid, Elhilu and Mahfouz found the highest satisfaction among interns in paediatrics and general surgery, with obstetrics the least favoured—almost the reverse of South African findings. Over 75% of Saudi interns reported feeling at least moderately prepared for the next stage of their careers. In Sweden, doctors complete a minimum 18-month internship before independent practice. Annual evaluations reveal both a strong sense of safety and support, though the 2023 survey also reported that 24% of junior doctors were considering leaving the profession [13–15].

Encouragingly, 84% of South African interns reported adequate supervision and support during their training, though there remains scope for improvement. The relative scarcity of published evaluations of internship adequacy worldwide, across diverse contexts, suggests a global need to strengthen research and policy attention towards the preparation of junior doctors.

### Limitations

This voluntary survey reflects the views of interns motivated to contribute, introducing potential sampling bias. Generalisability is limited by the relatively small sample size (*n* = 82) and by variation in exposure, experience, and support across hospitals nationally. Response rates also varied across questions, which may have influenced outcomes.

In South Africa, internship programmes are not centralised, and no public databases exist to establish the total number of eligible participants, limiting the accuracy of participation estimates. The questionnaire design also imposed constraints: some open-ended items lacked space for detailed responses, while the largely prescriptive format restricted opportunities for participants to elaborate on their perspectives. Additionally, the length of the survey may have deterred potential respondents.

Certain oversights on behalf of the authors further limit the findings, including the omission of orthopaedics from all questions and partial omission of paediatrics. These gaps may have excluded important insights into training adequacy.

Overall, while the study provides valuable perspectives on internship training, its findings should be interpreted cautiously, with recognition of the limitations inherent to cross-sectional survey design and the contextual variability of South African internship programmes.

### Recommendations

The authors recommend further, larger studies of a similar nature, incorporating interns from across the provinces of South Africa, possibly via a formal national enquiry. This should be done regardless of further plans for the internship period, but must be done if there is any discussion about changes to the internship programme. The findings of this study could also benefit from being further evaluated in a qualitative study aiming to elucidate specific opinions about the internship programme across stakeholders.

Furthermore, factors that make interns less likely to work in rural and primary care settings must continue to be investigated and addressed systematically, allowing for further service provision to the majority of the population. Efforts made to create a family medicine rotation that is fit-for-purpose should continue to be encouraged, while there is an opportunity for this rotation to incorporate aspects of rural healthcare that create pull factors for junior doctors to migrate to or stay in rural and primary care settings in their future practices.

Finally, we recommend that the internship programme not be shortened. From this data, the internship programme is meeting many of its stated goals, and interns value their time spent across disciplines. Most stakeholders think it is impossible to create competent junior doctors while decreasing the internship length.

## Conclusion

Medical interns constitute a critical component of South Africa’s health workforce, and optimising the internship programme is essential for sustaining future healthcare delivery. This survey indicates that the current two-year internship is largely effective in preparing interns for independent practice, except for the surgical rotation.

Policy-makers should treat internship structure as a matter of strategic importance and engage stakeholders before implementing changes, particularly regarding programme length. Maintaining strong supervision, adequate facility capacity, and robust opportunities for procedural and diagnostic skill development are essential. Placement in facilities that provide broad exposure across different levels of care, including urban and semi-urban contexts, should also remain a priority.

The divisive views on programme duration highlight the need for regular review. Re-evaluating the internship every two years, in consultation with stakeholders, would help ensure that its goals align with evolving healthcare needs while safeguarding the quality of training and patient care.

## Supplementary Information

Supplementary Material 1.

## Data Availability

The datasets used and/or analysed during the current study are available from the corresponding author on reasonable request.
