# Supplementary material for: Evaluating perspectives and attitudes towards the South African medical internship programme
Source: BMC Med Educ. 2025 Oct 10;25:1395. doi: 10.1186/s12909-025-07994-y (PMC12512394; doi:10.1186/s12909-025-07994-y)
Supplement: Supplementary file 1 — Supplementary Material 1. [file 12909_2025_7994_MOESM1_ESM.docx]

# Is the South African internship experience effective in achieving surgical system goals?

## **Consent form**

**Explanation of questionnaire**

We are a group of medical students from the University of Cape Town. This questionnaire is part of a study we are conducting in order to determine the attitudes and perceptions about the optimal duration of medical internship in South Africa for surgical and clinical skills training. We would like you to fill in a series of questions to help us with our study. We hope this provides us with data that can assist leadership around the country to design internship programmes optimally and gives you an opportunity to think about the kind of health professional training and experience you think is most valuable.

**Inclusion criteria** – you can partake in this questionnaire if you fall into any of the below groups of people

- Medical interns who are qualified doctors and are currently working for the government as an intern during either their first or second year of the internship period at a hospital affiliated to UCT or SU (see below for a list of inclusion hospitals)
- A community service doctor or medical officer that works as a doctor at a hospital affiliated to UCT or SU
- Internship curators at the hospitals that are connected to UCT or SU
- Heads of the following departments at hospitals that are connected to UCT or SU: Internal medicine, Surgery, Obstetrics, Paediatrics, Anaesthesia, Family medicine and Psychiatry

*For our purposes, the hospitals affiliated to either UCT or SU include the following: Groote Schuur Hospital; Victoria Hospital; New Somerset Hospital; Mitchell’s Plain District Hospital; Red Cross Children’s Hospital; Tygerberg Hospital; Karl Bremer Hospital; Khayelitsha District Hospital. There may be others, but those hospitals do not form part of this study population.*

**Confidentiality**

Please do not put your name on this questionnaire. We will collect your HPCSA number and email address to enter you into a random draw for prizes and to ensure that nobody takes the questionnaire more than once. Your email address and HPCSA numbers will be disaggregated from your answers. All the information you provide will only be accessed by the researchers, so your information is confidential and private. No one will know how you have answered these questions, so it is important that you answer carefully and honestly. There are no right or wrong answers and no good or bad answers. The questions are in reference to your experience and knowledge about the medical internship programme in South Africa. We want to fully understand your ideas, perceptions, and desires about the medical internship programme in South Africa.

You can leave the study at any time, however you will not be eligible for entry into the random draw for prizes unless you answer all questions in the questionnaire.

**Process**

This questionnaire has 4 sections:

1. Demographic information
2. Internship programme
3. Perception of a shortened internship programme
4. Impact on health system

# **Questionnaire**

## **Section 1: Demographics – for all study participants**

1. **What is your current job description?**

- 1st year Internship
- 2nd year Internship
- Community service doctor
- Medical officer
- Internship programme curator specify (medical officer, specialist)
- Head of Department specify department
- If HOD, please specify the department
- Other (specify)

1. **At which hospital are you currently situated?**

- Groote Schuur Hospital
- Tygerberg Hospital
- New Somerset Hospital
- Red Cross War Memorial Children’s Hospital
- Victoria Hospital
- Karl Bremer Hospital
- Mitchell’s Plain District Hospital
- Khayelitsha District Hospital
- Other (specify)

1. **Where did you do your undergraduate training?**

- University of Cape Town
- Stellenbosch University
- University of Witwatersrand
- University of Pretoria
- University of KwaZulu-Natal
- Sefako Makgatho Health Sciences University
- University of Free State
- Walter Sisulu University
- Non-South African university

If non-South African, specify in which country you did your training.

## **Section 2: Internship programme - only for current interns**

1. **What do you expect to gain from your internship time?** *(click all that apply)*

- A good salary with comfortable living options
- Appropriate induction into your role and the hospital you are placed in
- Surgical experience
- Diagnostic skills
- Opportunity to interact with seniors in ways that promote your learning and skills development
- Experience in prioritising patient safety
- Learning how to prioritise patient safety – specifically through teaching by and the example of clinical supervisors
- Relevant and helpful feedback from supervisors on a regular basis
- Immediate help from relevant supervisor should this be needed
- Experience and support in practising medicine that is evidence-based
- To be involved in or complete research projects
- Procedural skills
- A chance to build relationships with other health professionals
- Time management skills
- An opportunity to explore a new area of the country
- Access to subsidised accommodation (doctors’ quarters)
- A time of definite job security
- Confidence in managing patients independently
- More clinical responsibility
- Other (specify)

1. **Where would you prefer to spend the bulk of your internship time?** *(click all that apply)*

- Urban setting
- Peri-urban setting
- Rural setting
- A mix of the above

1. **What level of care would you prefer to spend the bulk of your internship practising in?**

- Primary care clinic
- District hospital
- Secondary level academic hospital
- Tertiary level academic hospital
- A mix of the above

1. **At which level of care do you see yourself spending the bulk of your future practising time in?**

- Primary care clinic
- District hospital
- Secondary level academic hospital
- Tertiary level academic hospital
- A mix of the above

Please provide a reason for this answer *(optional)*

1. **Where do you think practising during internship would make you best prepared for community service and independent practise?**

- Urban setting
- Suburban setting
- Rural setting
- A mix of the above

Please provide a reason for this answer *(optional)*

1. **Where do you currently feel most prepared to practice?**

- Primary care clinic
- District hospital
- Secondary level academic hospital
- Tertiary level academic hospital

1. **Where do you currently feel least prepared to practice?**

- Primary care clinic
- District hospital
- Secondary level academic hospital
- Tertiary level academic hospital

1. **Practising in which level of care during your internship would make you better prepared for community service and independent practise?**

- Primary care clinic
- District hospital
- Secondary level academic hospital
- Tertiary level academic hospital

Please provide a reason for this answer *(optional)*

## Section 2 – only for interns

1. **Have you begun or completed your surgical block during your internship?**

- Yes
- No

1. **If yes, do you feel like your surgical training and exposure during your internship time has been adequate for you to become an independent medical practitioner in South Africa?**

- Yes
- No

*Please elaborate (optional)*

1. **Have you begun or completed your anaesthesia block during your internship?**

- Yes
- No

1. **If yes, do you feel like your anaesthesia training and exposure during your internship time has been adequate for you to become an independent medical practitioner in South Africa?**

- Yes
- No

*Please elaborate (optional)*

1. **Have you begun or completed your obstetrics block during your internship?**

- Yes
- No

1. **If yes, do you feel like your obstetrics training and exposure during your internship time has been adequate for you to become an independent medical practitioner in South Africa?**

- Yes
- No

*Please elaborate (optional)*

1. **Do you feel like you have been adequately supported/supervised during your internship programme?**

- Yes
- No

1. **Which department below do you feel you had the most support or supervision?**

- Obstetrics and gynaecology
- Surgery
- Anaesthesia
- Medicine
- Family medicine
- Paediatrics
- Psychiatry

1. **Do you feel that the logbook requirements mandated for interns during their internship programme adequately addresses the public health needs of South Africa?**

- Yes
- Unsure
- No

*Please elaborate (optional)*

1. **Do you feel that the logbook requirements mandated for interns during their internship programme adequately prepares interns to become independent medical practitioners in South Africa?**

- Yes
- Unsure
- No

1. **If you were to improve the internship programme what would you change?**

*Full question*

Section 2: Internship programme - only for current interns

1. What do you expect to gain from your internship time? (click all that apply)

- A good salary with comfortable living options
- Appropriate induction into your role and the hospital you are placed in
- Surgical experience
- Diagnostic skills
- Opportunity to interact with seniors in ways that promote your learning and skills development
- Experience in prioritising patient safety
- Learning how to prioritise patient safety – specifically through teaching by and the example of clinical supervisors
- Relevant and helpful feedback from supervisors on a regular basis
- Immediate help from relevant supervisor should this be needed
- Experience and support in practising medicine that is evidence-based
- To be involved in or complete research projects
- Procedural skills
- A chance to build relationships with other health professionals
- Time management skills
- An opportunity to explore a new area of the country
- Access to subsidised accommodation (doctors’ quarters)
- A time of definite job security
- Confidence in managing patients independently
- More clinical responsibility
- Other (specify)

1. Where would you prefer to spend the bulk of your internship time? (click all that apply)

- Urban setting
- Peri-urban setting
- Rural setting
- A mix of the above

1. What level of care would you prefer to spend the bulk of your internship practising in?

- Primary care clinic
- District hospital
- Secondary level academic hospital
- Tertiary level academic hospital
- A mix of the above

1. At which level of care do you see yourself spending the bulk of your future practising time in?

- Primary care clinic
- District hospital
- Secondary level academic hospital
- Tertiary level academic hospital
- A mix of the above
- Please provide a reason for this answer (optional)

1. Where do you think practising during internship would make you best prepared for community service and independent practise?

- Urban setting
- Suburban setting
- Rural setting
- A mix of the above
- Please provide a reason for this answer (optional)

1. Where do you currently feel most prepared to practice?

- Primary care clinic
- District hospital
- Secondary level academic hospital
- Tertiary level academic hospital

1. Where do you currently feel least prepared to practice?

- Primary care clinic
- District hospital
- Secondary level academic hospital
- Tertiary level academic hospital

1. Practising in which level of care during your internship would make you better prepared for community service and independent practise?

- Primary care clinic
- District hospital
- Secondary level academic hospital
- Tertiary level academic hospital

Please provide a reason for this answer (optional)

**Section 2 – only for community service doctors and medical officers**

1. Do you feel like your surgical training and exposure during your internship time was adequate for you to become an independent medical practitioner in South Africa?

- Yes
- No
- Please elaborate (optional)

1. Do you feel like your anaesthesia training and exposure during your internship time was adequate for you to become an independent medical practitioner in South Africa?

- Yes
- No
- Please elaborate (optional)

1. Do you feel like your obstetrics training and exposure during your internship time was adequate for you to become an independent medical practitioner in South Africa?

- Yes
- No
- Please elaborate (optional)

1. Do you feel like you were adequately supported/supervised during your internship programme?

- Yes
- No

1. Which department below do you feel you had the most support or supervision during your internship time?

- Obstetrics and gynaecology
- Surgery
- Anaesthesia
- Medicine
- Family medicine
- Paediatrics
- Psychiatry

1. Do you feel that the logbook requirements mandated for interns during their internship programme adequately addresses the public health needs of South Africa?

- Yes
- Unsure
- No

Please elaborate (optional)

1. Do you feel that the logbook requirements mandated for interns during their internship programme adequately prepares interns to become independent medical practitioners in South Africa?

- Yes
- Unsure
- No

1. If you were to improve the internship programme what would you change?

- Full question

## **Section 3: Perceptions of a shortened internship period and its effect on surgical training and exposure – only for interns, community service doctors and medical officers**

1. **What do you think is the ideal length of the internship period?**

- One year
- Two years
- Three years
- Unsure

1. **What possible benefits do you think there may be if the internship period was changed to a truncated 1-year internship programme? *Please be in-depth.***

- Worded answer

1. **Do you think any of the following situations are possibilities if the internship period is shortened to a single year in duration?** *(click any that apply)*

- No
- Interns will have more freedom of choice in living situation
- Interns will haver quicker transition to independent medical practice
- Interns will have less mandatory time in the public sector
- Interns will have less time before registrar training
- Other – *please specify, as in depth as possible*

Please add any additional thoughts, suggestions or concerns

If yes is clicked for any of the above, the following question will be asked to the participant: Do you think this would be a benefit of a shortened internship period or a flaw in a shortened internship period?

1. **What other possible outcomes do you envision coming into fruition if the internship period were to be shortened to a single year duration?**

- Worded answer

1. **Please order the below options into what you think should be the top 5 priorities for the internship period.**

- A good salary with comfortable living options
- Appropriate induction into your role and the hospital you are placed in
- Surgical experience
- Diagnostic skills
- Opportunity to interact with seniors in ways that promote your learning and skills development
- Experience in prioritising patient safety
- Learning how to prioritise patient safety – specifically through teaching by and the example of clinical supervisors
- Relevant and helpful feedback from supervisors on a regular basis
- Immediate help from relevant supervisor should this be needed
- Experience and support in practising medicine that is evidence-based
- To be involved in or complete research projects
- Procedural skills
- A chance to build relationships with other health professionals
- Time management skills
- An opportunity to explore a new area of the country
- Access to subsidised accommodation (doctors’ quarters)
- A time of definite job security
- Confidence in managing patients independently
- More clinical responsibility
- Other (specify)

1. **Please rank the following disciplines in order from first priority to last in terms of the following: In which disciplines do you most desire *exposure to* during your internship period?**

- Medical disciplines (internal medicine, cardiology, pulmonology etc)
- Surgical disciplines (general surgery, trauma surgery)
- Paediatric disciplines (neonatal care, paediatric medicine or paediatric surgery)
- Obstetric and gynaecological care
- Anaesthesia care
- Family medicine
- Psychiatric care

1. **Please rank the following disciplines in order from first priority to last in terms of the following: In which disciplines do you most desire *training in* during your internship period?**

- Medical disciplines (internal medicine, cardiology, pulmonology etc)
- Surgical disciplines (general surgery, trauma surgery)
- Paediatric disciplines (neonatal care, paediatric medicine or paediatric surgery)
- Obstetric and gynaecological care
- Anaesthesia care
- Family medicine
- Psychiatric care

1. **Please rank the following disciplines according to which disciplines you think should be mandatory during the internship period, if the main goal is to *improve the health of the population.***

- Medical disciplines (internal medicine, cardiology, pulmonology etc)
- Surgical disciplines (general surgery, trauma surgery)
- Paediatric disciplines (neonatal care, paediatric medicine or paediatric surgery)
- Obstetric and gynaecological care
- Anaesthesia care
- Family medicine
- Psychiatric care

1. **Please rank the following disciplines according to which disciplines you think should be mandatory during the internship period, if the main goal is to *develop medical practitioners who are ready for independent practise in South Africa.***

- Medical disciplines (internal medicine, cardiology, pulmonology etc)
- Surgical disciplines (general surgery, trauma surgery)
- Paediatric disciplines (neonatal care, paediatric medicine or paediatric surgery)
- Obstetric and gynaecological care
- Anaesthesia care
- Family medicine
- Psychiatric care

1. **Do you think a shortened internship will have a negative impact on your surgical training?**

- No
- Maybe
- Yes --- if yes then follow up question: Would you be willing to compromise your surgical training in exchange for a shortened internship period? Answer options: yes, unsure, no

1. **Do you think a shortened internship will have a negative impact on your obstetrics training?**

- No
- Maybe
- Yes -- if yes then followup question: Would you be willing to compromise your obstetrics training in exchange for a shortened internship period? Answer options: yes, unsure, no

1. **Do you think a shortened internship will have a negative impact on your medical training?**

- No
- Maybe
- Yes -- if yes then followup question: Would you be willing to compromise your medical training in exchange for a shortened internship period? Answer options: yes, unsure, no

1. **Do you think that there would be any harms associated with a shortened internship period?**

- Yes
- Unsure
- No

1. **If yes to the above, please list in detail possible harms you can envision due to a shortened internship period.**
2. **What harms do you think would be associated with a shortened internship period?**

- Inadequate teaching time
- Decreased mentorship opportunities
- Job security for less time
- Decreased clinical exposure
- Decreased chance of doing research
- Increase in competition in private sector
- Increased competition for further training

Please add any additional thoughts, suggestions or concerns

1. **Do you think a shortened internship period should be optional? i.e. should prospective interns be able to choose between a one- and two-year internship?**

- Yes
- Yes, but only if there is a valid reason to choose a shortened period – academic advancement, family emergencies
- No
- Not sure

1. **Do you think that there should be flexibility in internship training in terms of what rotations and specialties you choose to complete in 1 or 2 years and for what lengths?**

- Yes
- Not sure
- No

Please explain your answer *(optional)*

1. **Do you think the possible benefits of a shortened internship outweigh the possible disadvantages?**

- Yes
- Maybe
- No

1. **Do you think a one-year internship period is preferable to a two-year period on a national, mandated level?**

- Definitely not
- There are benefits, but not enough to warrant this change
- Either is acceptable
- Benefits probably outweigh potential harms
- Definitely

Please explain your answer to the previous question

1. **Do you think a truncated internship period would lead to the general public having mistrust in the health system or competency of doctors?**

- Yes
- Unsure
- No
- Yes, unless specific information and campaigning is done to allay fears

1. **Do you think a 1-year internship would compromise patient care during the internship period?**

- Yes
- Unsure
- No

1. **Do you think a 1-year internship would compromise patient care by interns post-internship?**

- Yes
- Unsure
- No

1. **Do you think shortening the period to one year would result in greater supervision of interns as they provide patient care?**

- No
- Maybe
- Yes

1. **Do you think a one-year internship period is preferable to a two-year period?**

- Definitely not
- There are benefits, but not enough to warrant this change
- Either is acceptable
- Benefits probably outweigh potential harms
- Definitely

Please explain your answer to the previous question

1. **Do you have any suggestions to improve the current system?**

- No
- Yes

If yes, please elaborate *here (optional)*

1. **Do you think a one-year internship gives intern the minimum competencies required for surgical care?**

- Yes
- Maybe
- No

1. **Do you think a one-year internship gives intern the minimum competencies required for obstetric care?**

- Yes
- Maybe
- No

1. **Do you think a one-year internship gives intern the minimum competencies required for anaesthesia care?**

- Yes
- Maybe
- No

1. **Do you think a one-year internship gives intern the minimum competencies required overall patient care as an independent medical practitioner in South Africa?**

- Yes
- Maybe
- No

1. **Would you, personally, prefer a one- or two-year internship period?**

- One
- Two

## **Questions for internship curators and heads of departments**

1. **What do you think interns *desire* to gain from their internship time?**

- A good salary with comfortable living options
- Appropriate induction into your role and the hospital you are placed in
- Surgical experience
- Diagnostic skills
- Opportunity to interact with seniors in ways that promote your learning and skills development
- Experience in prioritising patient safety
- Learning how to prioritise patient safety – specifically through teaching by and the example of clinical supervisors
- Relevant and helpful feedback from supervisors on a regular basis
- Immediate help from relevant supervisor should this be needed
- Experience and support in practising medicine that is evidence-based
- To be involved in or complete research projects
- Procedural skills
- A chance to build relationships with other health professionals
- Time management skills
- An opportunity to explore a new area of the country
- Access to subsidised accommodation (doctors’ quarters)
- A time of definite job security
- Confidence in managing patients independently
- More clinical responsibility
- Other (specify)

1. **What do you think interns should gain from their internship time? (click all that apply)**

- A good salary with comfortable living options
- Appropriate induction into your role and the hospital you are placed in
- Surgical experience
- Diagnostic skills
- Opportunity to interact with seniors in ways that promote your learning and skills development
- Experience in prioritising patient safety
- Learning how to prioritise patient safety – specifically through teaching by and the example of clinical supervisors
- Relevant and helpful feedback from supervisors on a regular basis
- Immediate help from relevant supervisor should this be needed
- Experience and support in practising medicine that is evidence-based
- To be involved in or complete research projects
- Procedural skills
- A chance to build relationships with other health professionals
- Time management skills
- An opportunity to explore a new area of the country
- Access to subsidised accommodation (doctors’ quarters)
- A time of definite job security
- Confidence in managing patients independently
- More clinical responsibility
- Other (specify)

1. **Please order the below options into what you think should be the top 5 priorities for the internship period.**

- A good salary with comfortable living options
- Appropriate induction into your role and the hospital you are placed in
- Surgical experience
- Diagnostic skills
- Opportunity to interact with seniors in ways that promote your learning and skills development
- Experience in prioritising patient safety
- Learning how to prioritise patient safety – specifically through teaching by and the example of clinical supervisors
- Relevant and helpful feedback from supervisors on a regular basis
- Immediate help from relevant supervisor should this be needed
- Experience and support in practising medicine that is evidence-based
- To be involved in or complete research projects
- Procedural skills
- A chance to build relationships with other health professionals
- Time management skills
- An opportunity to explore a new area of the country
- Access to subsidised accommodation (doctors’ quarters)
- A time of definite job security
- Confidence in managing patients independently
- More clinical responsibility
- Other (specify)

1. **Please detail anything that you think interns should gain from their internship period that was not mentioned above (optional and long answer)**
2. **Do you think the duration of the internship programme affects competency of future independent doctors?**

- Yes
- No
- Maybe

*If yes, why?*

1. **Do you think a one-year internship period is preferable, as a national mandated time period, instead of a two-year period?**

- No
- Unlikely
- Equally preferable
- Likely
- Definitely

1. **What benefits do you expect there to be from a shortened internship period?**

- More freedom of choice in living situation for interns
- Quicker transition to independent medical practice for interns
- Less mandatory time in the public sector for interns
- Less time before registrar training
- Increased research output from early-career clinicians
- None
- Other

Please add any additional thoughts, suggestions or concerns

1. **Do you think there would be any harms associated with a shortened nationally-mandated internship period of a single year?**

- Yes. If yes, please list as many as you can and in as much detail as possible.
- Maybe
- No

1. **Do you think that any of the below may be associated with a nationally-mandated shortened internship period?**

- None – interns can gain the same skills from a shortened internship programme
- Inadequate teaching time
- Decreased mentorship opportunities
- Decreased job security
- Decreased clinical exposure
- Decreased surgical skills
- Decreased competency and confidence in managing emergencies
- Increased morbidity and mortality for patients in the future
- Increase in competition in private sector
- Increased competition for further training
- For each yes bullet point ticked, ask: ‘Do you think this would be a benefit or a harm for the health system?’ with ‘Yes, Maybe, No’ as possible answers to this question.

Please add any additional thoughts, suggestions or concerns

1. **Do you think the possible benefits of a shortened internship outweigh the possible disadvantages?**

- Yes. If yes, please briefly explain your answer
- Maybe
- No

1. **Do you think a 1-year internship would compromise intern training?**

- Yes
- Unsure
- No

1. **Do you think a 1-year internship would compromise intern *surgical* training?**

- Yes
- Unsure
- No

1. **Do you think a 1-year internship would compromise intern *internal medicine* training?**

- Yes
- Unsure
- No

1. **Do you think a 1-year internship would compromise intern *obstetric* training?**

- Yes
- Unsure
- No

1. **Do you think a 1-year internship would compromise intern *paediatric* training?**

- Yes
- Unsure
- No

1. **Do you think a 1-year internship would compromise intern *anaesthesia* training?**

- Yes
- Unsure
- No

1. **Do you think a 1-year internship would compromise intern *psychiatry* training?**

- Yes
- Unsure
- No

1. **Do you think a 1-year internship would compromise intern *family medicine* training?**

- Yes
- Unsure
- No

1. **Do you think a 1-year internship would compromise patient care during the internship period?**

- Yes
- Unsure
- No

1. **Do you think a 1-year internship would compromise patient care by interns post-internship?**

- Yes
- Unsure
- No

1. **Do you think shortening the period to one year would result in greater supervision of interns as they provide patient care?**

- No
- Maybe
- Yes

1. **Do you think a one-year internship period is preferable to a two-year period?**

- Definitely not
- There are benefits, but not enough to warrant this change
- Either is acceptable
- Benefits probably outweigh potential harms
- Definitely

Please explain your answer to the previous question

1. **Do you think a truncated internship period would lead to the general public having mistrust in the health system or competency of doctors?**

- Yes
- Unsure
- No
- Yes, unless specific information and campaigning is done to allay fears

1. **Do you have any suggestions to improve the current system?**

- No
- Yes

If yes, please elaborate *here (optional)*

1. **Do you think a one-year internship gives intern the minimum competencies required for surgical care?**

- Yes
- Maybe
- No

1. **Do you think a one-year internship gives intern the minimum competencies required for obstetric care?**

- Yes
- Maybe
- No

1. **Do you think a one-year internship gives intern the minimum competencies required for anaesthesia care?**

- Yes
- Maybe
- No

1. **Do you think a one-year internship gives intern the minimum competencies required overall patient care as an independent medical practitioner in South Africa?**

- Yes
- Maybe
- No

## **Section 4: Impact on health system**

1. **How do you expect the public to react to news that the internship period is being shortened, if this were to happen?**

- Will disapprove the changes
- Will be neutral about the changes
- Would celebrate the changes

Please provide a reason for your answer *(optional)*

1. **How do you expect medical practitioners to react to news that the internship period is being shortened, if this were to happen?**

- Will disapprove the changes
- Will be neutral about the changes
- Would celebrate the changes

1. **Do you think that a truncated internship period would place more pressure on hospital and intern managers?**

- Yes
- Unsure
- No

Please provide a reason for your answer *(optional)*

1. **Do you think that a truncated internship period would place undue pressure on interns?**

- Yes
- Unsure
- No

Please provide a reason for your answer *(optional)*

1. **Do you think that a shortened internship would affect patient load at affected hospitals?**

- Yes
- Unsure
- No

1. **Do you think that a shortened internship would affect patient care and management at affected hospitals?**

- Yes
- Unsure
- No

1. **Do you think that interns that complete a single-year internship period will be unsafe medical practitioners?**

- Yes
- Unsure
- No
